# Diffractive Optical Analysis for Refractive Index Sensing using Transparent Phase Gratings

**DOI:** 10.1038/srep16687

**Published:** 2015-11-18

**Authors:** Nityanand Kumawat, Parama Pal, Manoj Varma

**Affiliations:** 1 Center for Nano Science and Engineering, Indian Institute of Science, Bangalore; 2 Robert Bosch Center for Cyber Physical Systems, Indian Institute of Science, Bangalore; 3 Dept of Electrical Communication Engineering, Indian Institute of Science, Bangalore

## Abstract

We report the implementation of a micro-patterned, glass-based photonic sensing element that is capable of label-free biosensing. The diffractive optical analyzer is based on the differential response of diffracted orders to bulk as well as surface refractive index changes. The differential read-out suppresses signal drifts and enables time-resolved determination of refractive index changes in the sample cell. A remarkable feature of this device is that under appropriate conditions, the measurement sensitivity of the sensor can be enhanced by more than two orders of magnitude due to interference between multiply reflected diffracted orders. A noise-equivalent limit of detection (LoD) of 6 × 10^−7^ was achieved with this technique with scope for further improvement.

A large body of literature has been devoted to the design and application of optical biosensors that operate in labeled (typically, fluorescence-based) as well as label-free detection formats^1^,^2^,^3^. Both embodiments offer complementary information regarding the structure and interactions of biomolecules of interest. Label-free sensing mechanisms offer reduced complexity and costs by obviating the need for tedious sample processing steps in the detection protocol. A variant of a sensing transduction scheme that has garnered significant attention in recent years is based on the use of diffraction gratings for detecting changes in the refractive index of the analyte by inspecting the far-field^4^,^5^,^6^,^7^,^8^,^9^,^10^,^11^.

We recently demonstrated a detection and analysis device based on a silicon dioxide (SiO_2_) layer on a silicon substrate where the planar oxide layer was modified to a micro-structured grating for controlling light-matter interactions to yield necessary information about the target sample^12^. This sensor design, referred to as a Diffractive Interference Sensor (DIS) in the aforementioned paper^12^, is hereafter referred to as a Diffractive Optical Analyzer (DiOpter). The benefits of sensing based on the DiOpter over other mechanisms^13^,^14^,^15^,^16^,^17^,^18^ include the ability to perform, real-time refractive index data acquisition enabling distinction between surface reactions, important for bio-diagnostic applications, from bulk changes based on differences in the time-scales of these processes. Real-time diffraction measurements also provide statistically relevant, time-resolved information about surface reaction kinetics, for instance, to extract affinity constants and disassociation constants of surface immobilized receptor-ligand interactions, by averaging over multiple diffracting sites/centers^4^. In addition, the effects of thermal and flow fluctuations in the measured signal are mitigated to a great extent since all diffracted orders are equally affected by any systemic drifts^12^. Previously reported implementations based on silicon substrates at visible wavelengths only allowed for operation in the reflection mode, which is not very amenable to miniaturization. Transmission measurements can potentially allow compact system configurations, for instance as a snap-on device on top of smartphone cameras. Therefore, we theoretically investigated the performance of phase gratings in transparent substrates, such as glass, for the measurement of bulk and surface refractive indices and found that under appropriate circumstances the sample fluid layer thickness can effectively amplify the refractive index sensitivity by up to two orders of magnitude. In this article we provide a detailed theoretical description and analysis along with pertinent experimental results to demonstrate interferometric surface and bulk refractive index sensing using pre-patterned glass ‘chips’, both in the reflection mode (R-mode) as well as in the transmission mode (T-mode). Other substrates which are transparent in the visible wavelength range, such as thermo-plastics, can also be employed for the DiOpter, which potentially allow for mass production of these sensors using hot-embossing or similar molding processes. In the subsequent sections, we describe in greater detail, the structure of the DiOpter device and the signal measurement process.

The basic operational principle of the DiOpter system, shown in Fig. 1, is the differential response of the individual orders constituting the observed far-field pattern to bulk or surface refractive index changes (depending on the sensor configurations which will be discussed below). The differential response is used to suppress thermal or flow induced fluctuations in the sample cell by up to 40x as shown previously^12^. Our proposed model, based on the multi-beam interference (MBI) phenomenon, describes bulk refractive index sensing in R-mode as well as T-mode. In our experiments, we were able to achieve an experimental RI (refractive index) limit of detection (LoD) of around 6 × 10^−7^ RIU (Refractive Index Unit) in the R-mode, making it comparable to some of the best refractive index sensing performances reported in literature^19^,^20^,^21^, although, a direct comparison is often challenging owing to the variations in the experimental conditions and sensitivity definitions^11^.

## Results

To characterize the DiOpter bulk refractive index detection sensitivity, we used solutions of NaCl-DI water mixtures with varying NaCl molar concentrations ranging from 0 to 0.5 M corresponding to refractive indices ranging from 1.333 (0M) to 1.338 (0.5 M). A baseline for the measurements was established by using the 0M sample (RI = 1.333) in the flow cell. The sample concentration was varied sequentially by 0.1 M till it reached 0.5 M, after which, the initial signal was recovered by using DI water (0M) in the sample cell. The refractive indices of the stock solutions were measured independently with a commercial bench-top refractometer (Anton Paar Abbemat 200) yielding values in agreement with previously reported values in literature^22^,^23^. We recorded the response of the observed zeroth and first order intensities, denoted by I_0_ (I_0,0_ in the 2D diffraction pattern) and I_1_ (I_0,1_ in the 2D diffraction pattern) respectively, in both the R- (Fig. 2a) and T- (Fig. 2b) modes, to changes in the refractive index of the sample in the flow cell. The arrows in the graph indicate time points at which the sample concentration was changed. The DiOpter responded linearly to RI changes in both cases [Fig. 2(c)]. As seen from Fig. 2(a,b), the ratio (I_1_/I_0_) eliminates any systemic drifts (laser fluctuations or any temperature or flow perturbances) since they affect these orders equivalently. This ratiometric approach suppressed signal variations due to inherent system fluctuations by a factor of about 40 based on the comparison of the magnitude of signal variation in the first order and the ratio channels. Drift compensation using the ratiometric scheme is clearly demonstrated in the 0.3 M step in Fig. 2 (a) where the detected first order signal I_1_ undergoes a significant drift which is compensated by taking the I_1_/I_0_ ratio. The intensity of the zeroth order was observed to be fairly invariant to RI changes, which is consistent with our proposed theoretical model described later. The limit of detection (LoD), i.e., the smallest selectable RI change, was determined by reducing the NaCl concentrations in the stock solution (0.8 mM, 1 mM, 2 mM, 4 mM and 6 mM) and measuring the sensor response in the R-mode. The 0.8 mM to 1 mM step represents a refractive index change of about 2 × 10^−6^ RIU, which is clearly distinguishable. The noise-equivalent LoD (corresponding to signal-noise ratio equal to 1) was estimated to be approximately 6 × 10^−7^ RIU by dividing the standard deviation of the measurement by the slope of the linear fit shown in Fig. 2 (d). As pointed out in the introduction section, the LoD is among some of the best LoD performances described in literature. For instance, typical bench-top SPR systems achieve a LoD of about 1 × 10^−6^RIU while phase sensitive SPR achieves a LoD of about 10^−7^ RIU^21^,^24^.

A vast majority of biosensing applications rely on the measurement of surface adsorption of target molecules onto a functionalized sensor surface. To demonstrate the capability of the DiOpter for detecting molecular adsorption processes occurring on the device surface, we used polyelectrolyte molecules, which provide a well-calibrated thin film system capable of adhering to almost any substrate independent of the surface chemistry^25^. Polyelectrolytes are charged polymer species, which electrostatically adsorb on surfaces exhibiting opposite charge in a self-limiting assembly process^26^. By alternately exposing a substrate to cationic and anionic polyelectrolytes, it is possible to create polyelectrolyte multilayers through electrostatic LbL (layer-by-layer) assembly. We used cationic polyelectrolyte poly(allylamine hydrochloride) (PAH) and anionic polyelectrolyte poly (acrylic acid) (PAA) to form 8 bilayers on the DiOpter surface with DI water rinse steps in between to remove loosely bound molecules. Fig. 3 (a) shows the experimentally observed diffraction intensities I_0_ and I_1_ as a function of the polyelectrolyte LbL build-up process. We repeated the polyelectrolyte LbL assembly on three separate devices to ascertain the robustness of surface adsorption measurements with the DiOpter. As shown in Fig. 3(b), the repeatability of the measurements are within 2–3%. We used the MBI model to fit the observed experimental response to parameters of the adsorption process. A thickness growth curve represented by *d*_*n*_ = 0.35*n*^2^, was used to estimate *d*_*n*_, which is the thickness of the multilayer after *n* layers^27^. The refractive indices of PAH and PAA were taken as 1.49 and 1.47 respectively. These values are well within the range of effective refractive index values reported for PAH/PAA multilayers^28^,^29^,^30^. The difference in refractive indices between these polymers results in the “sawtooth” pattern (Fig. 3(b)) which has also been previously reported in some studies^31^,^32^. Using the time-resolved data collected by the DiOpter sensor, we estimated the kinetic parameters of polyelectrolyte surface adsorption by fitting the signal to the first order (exponential) kinetic adsorption or desorption curves as shown in Fig. 3(c,d). A single exponential function of the form y = *A* + *Be*^−*kt*^ was found to be a good fit to the experimental data as opposed to a double exponential function reported in previous studies on other polyelectrolyte systems^33^,^34^. This fitting function gave the characteristic time scale (1/*k*) of PAH adsorption to the surface of about 43 seconds (*k* = 0.023 s^−1^). Although, to the best of our knowledge, there are no previously reported values of the characteristic time scales of PAH or PAA adsorption to glass, the value reported here is close to those reported for other polyelectrolyte systems^33^,^34^,^35^. A similar fitting procedure for the DI water rinse step resulted in a characteristic time scale of about 6 seconds indicating a rapid signal response, as expected from a bulk change in the refractive index. Thus, this data demonstrates the possibility of distinguishing surface and bulk RI changes based on the characteristic time scale of the response, i.e. bulk changes occur in diffusion time-scales and the surface interactions occur in a combination of diffusion plus reaction time-scales which is generally longer.

## Mathematical Modeling and Discussion

The data shown in Figs 2 and 3 demonstrate the ability of the DiOpter system to measure bulk refractive index as well as surface adsorption in a time-resolved manner while the ratiometric analysis compensates for system drifts. A salient feature of the data presented in Fig. 2 is the higher bulk refractive index sensitivity of the R-mode compared to the T-mode. In order to understand this effect, we modeled the DiOpter performance on the basis of a Multi Beam Interference (MBI) model as shown in Fig. 4. The detected first order signal in either R- or T-mode can be written as the superposition of three field components as shown in Fig. 4,


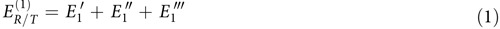


The detected intensity is the squared modulus of this field and as shown in detail in the Methods section and supplementary document, the intensity of the first order in the R-mode can be written as


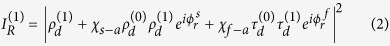


and, similarly for the T-mode,


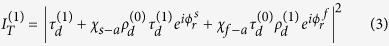


Here, *ρ*_*d*_ and *τ*_*d*_ refer to the reflected and transmitted diffraction efficiencies with the superscript in brackets denoting zero (0,0) or first (1,0) order; 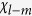 is the Fresnel reflection coefficient with *l* and *m* denoting the interfaces labeled as *a* (*a*ir), *s* (glass substrate) and *f* (*f*luid) and the phase factors are given by


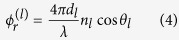


with *l* = *s* (glass substrate) or *f* = (fluid) and *d, n, θ* and *λ* representing the thickness, refractive index, incident angle and wavelength respectively. We numerically evaluated Eq (2) and (3) using a MATLAB code. An examination of the numerical order of the terms in Eq. (2) and (3), as shown in the supplementary document (Figures S1 and S2), allows us to make some simplifications to develop insights into DiOpter measurement sensitivity. Equations (2) and (3) simplify to


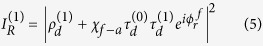


and,


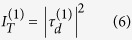


The difference in the terms contained in Eq (5) and (6) is the origin of the different index sensitivities in R and T-modes, as explained below.

Choosing 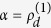 and 
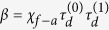
 we can write Eq. (6) as,


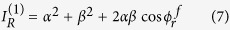


This is the well-recognized expression for two-beam interference.

We define the sensitivity of measurements as the relative signal change per unit refractive index change of the sample 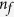 which can be written as,


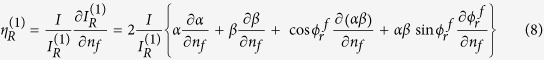


Inserting the expression for 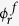 from Eq. (4) in the last term in Eq. (8) leads to,


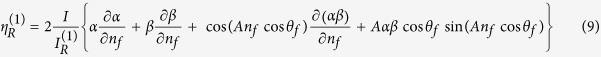


where,


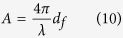


In a similar manner, the sensitivity in transmission mode is given by,


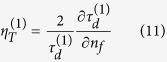


The factor *A* in the expression for the reflected mode sensitivity (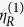), essentially acts as an amplification term for an index change o_f_ *n*_f_ when 
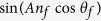
 approaches unity. This is because under this condition, the term involving A in Eq. (9) becomes the dominant term as the fluid depth is typically of the order of 10–100 microns which is substantially larger than the wavelength (See supplementary Fig. S3). Thus, by choosing an appropriate operating point, the R-mode sensitivity can be enhanced by increasing fluid layer thickness *d*_*f*_ as seen from Eq. (9). As shown in the supplementary document (Fig. S4), the amplified sensitivity in R-mode can be as high as about 700/RIU for a fluid layer depth of about 50 microns. Larger amplification factors can be achieved with even larger *d*_*f*_ but requires increasingly precise alignment of the incident angle due to rapid variation of the 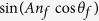 term with larger *A*. However, if the 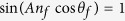 condition can be attained robustly along with large *d*_*f*_ values, the sensitivity of the R-mode measurement can significantly exceed the performance of many of the techniques described in literature^19^,^20^,^21^. By choosing a different set of material and geometric parameters, one can obtain a similar enhancement for T-mode measurements as well. In addition, we would like to clarify that although the discussion presented above is based on the measurement of bulk refractive measurement, the amplification factor A would also apply for surface adsorption as the signal is still resulting from interferometric mixing of zeroth and first orders as described in Eq. (5). In other words, one can consider the case of surface adsorption as by treating the fluid layer and the adsorbed layer as a composite medium with an effective index.

In the analysis above, we have only considered the variations in the observed first order and this is because the zeroth order, in reflection as well as in transmission, is relatively unperturbed by small changes in refractive index. This can be readily inferred from Fig. 2(a,b). This can be understood by noting that the detected zero order in the case of R-mode measurement consists of interference between the specularly reflected beam at the air-glass interface and the efficiency of the diffracted zeroth order 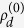. For our system, the Fresnel reflection coefficient for specular reflection is about 30 times stronger than 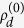 (Supplementary document), therefore the effect of 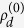 on the detected zeroth order signal in the R-mode can be neglected. As the specular reflection at the air-glass interface does not depend on *n*_*f*_, the detected zero order signal also remains invariant to *n*_*f*_ so that the observed change in the I_1_/I_0_ signal is only due to the change in the first order, which justifies our analysis. The theoretical treatment presented above is valid for bulk refractometric sensing as well sensing of surface adsorption by appropriately calculating the *r*(*x,y*) or *t*(*x,y*) in Eq. (1) and (3) using a transfer matrix method for multilayer films^36^.

From Eq. (7), we see that the MBI model predicts a periodic modulation of the detected first order intensity in the R-mode with respect to the incident angle. Inserting 
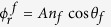
in Eq. (7), we can approximate the periodicity ∆*θ* to be 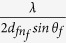, which works out to be about 0.7 degrees for our experimental system. Figure 5(a) shows the experimentally obtained variation of the detected first order intensity as a function of the incident angle. In order to compare the experimental data to the numerical calculation, the detected first order intensity averaged over *θ*_*f*_ was normalized for the experimental as well as the simulated data. From Fig. 5 (a) we see that the MBI model matches the periodicity and the interferometric modulation depth^36^ of the experimental data quite well. The experimentally obtained periodicity of the interferometric modulation of about 0.5 degrees is close to the 0.7 degrees approximated earlier. The amplification factor (A) in the MBI model (Eq. (10)) leads to a significantly higher sensitivity for R-mode measurements compared to T-mode measurements over a wide range of grating depths (*d*_*g*_). This is experimentally validated by the data presented in Fig. 2(a,b) where the T-mode signal change is much smaller than the corresponding R-mode signal change (higher NaCl concentrations were used in the T-mode measurements to illustrate this point in Fig. 2(a,b)). We quantitatively compared the R- and T-mode sensitivities across devices with different grating depths using NaCl -DI water solutions of varying concentrations. Numerical estimation of the sensitivities using the MBI model was done by calculating the difference in the first order signal corresponding to a refractive index change of 10^−3^ RIU. The experimentally measured R-mode index sensitivity was about an order of magnitude higher than the corresponding T-mode measurements, as shown in Fig 5(b), which was close to that predicted by the MBI model. Finally, the MBI model predicted an increase in the I_1_/I_0_ signal with increasing *n*_*f*_ for R-mode measurements (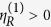, supplementary Figure S4) and a decrease for the corresponding case in the T-mode (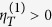, supplementary Figure S4). The experimental data shown in Fig. 2(a,b) confirms this behavior in accordance with the MBI model.

In summary, we have demonstrated a bulk and surface refractive index sensor based on the interferometric mixing of multiply reflected diffraction orders from a 2D grating. As shown in the preceding sections, the behavior predicted by the MBI model is in good agreement with the experimental data. The ratiometric approach eliminates source intensity noise and other drifts in the system. The LoD of the sensor is around 1.8 × 10^−6^ RIU which makes it comparable to well-established techniques among the state of the art. By exploiting the amplification factor arising out of multi-beam interference, the LoD can be improved further. The sensor is also capable of resolving the kinetics of surface adsorption processes, which is useful for discriminating between bulk and surface index perturbations. In addition, operating the device in transmission permits compact system designs which could, for instance, be integrated with smartphone cameras for applications such as handheld point-of-care systems for biochemical analyses.

## Methods

### Sensor Fabrication

**Figure 1:**
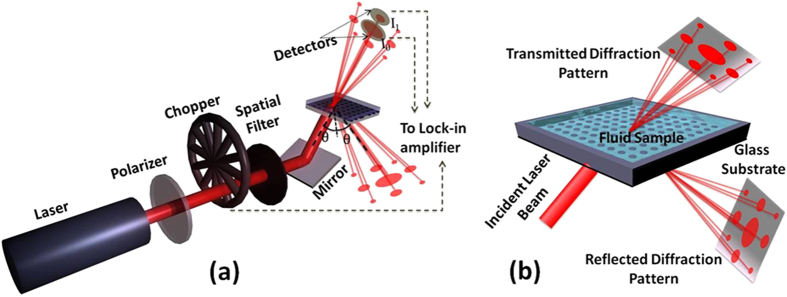
Schematic of the DiOpter sensor and the measurement setup, (a) showing the optical layout for R- and T-mode measurements and (b) shows a close-up view of the sensor consisting of micro-patterned structures on a glass substrate producing a diffraction pattern on illumination with a 633 nm He-Ne laser.

**Figure 2:**
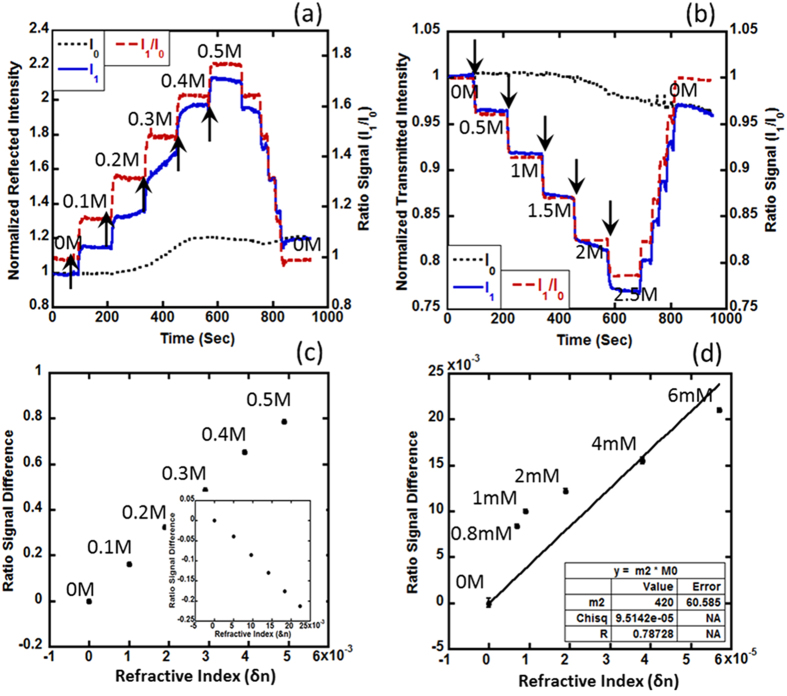
NaCl-DI water mixtures with varying salt content were introduced inside the sample cell at instances indicated by the black arrows. Drift compensation by ratioing the zeroth and first order diffraction intensities is apparent (0.3 M step in (**a**)) in R-mode as well as in the T-mode (2 M step in(**b**)). The response of the sensor in this range was roughly linear as shown in (**c**) for both R (main figure) and T-mode (inset) measurements. The limit of detection (LoD) was estimated for R-mode measurements using NaCl-DI water mixtures of lower concentration (**d**). The noise-equivalent LoD was found to be around 6 × 10^−7^ RIU.

The DiOpter sensor depicted in Fig 4, consists of a two-dimensional microarray of 10 μm-diameter circular pillars on a glass substrate, which forms a diffraction grating. The diffraction grating was fabricated photolithographically with 10-μm periodicity in a double-sided mask aligner (EVG 620). A chrome mask was used for transferring the circular patterns by using a positive tone photoresist S1813 (Shipley) on the glass cover slide. After UV exposure in the mask aligner, the devices were developed in MF-26A developer solution (Shipley). The developed devices were subsequently etched in a buffered HF solution (nominal etch rate of approximately 50 nm per minute) to achieve the desired grating depth *d*_*g*_, as shown in Fig. 4(a). The grating depth of the devices used for refractive index measurements ranged from 50 nm to 450 nm. A flow cell was constructed using a 50-μm-thick silicone O-ring covered with an acrylic sheet containing the sample inlet and outlet. Samples were injected into the flow cell through Teflon tubes connected to a syringe pump. For the optical detection (Fig. 1(a)), collimated light from a 633-nm He-Ne laser (Thorlabs HNL 050L) was passed through a polarizer and modulated with a mechanical chopper. The beam was incident on the sensor through the bottom surface at the desired angle (Fig. 1(b)). Intensity patterns were observed in reflection as well as in transmission. The intensities of the zeroth (0,0) and first order (0,1) were balanced using neutral-density filters before impinging on two identical Si photo-detectors (Thorlabs DET100A/M), whose outputs were sent to two SR-830 lock-in amplifiers (Stanford Research Systems).

## MBI Model

The DiOpter sensor is essentially a phase grating fabricated on the glass substrate. The DiOpter surface can be modeled as a 2D periodic modulation of reflection *r*(*x,y*) (Fig. 4)


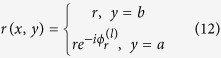


where,


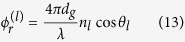


Here, *l* = *f* or *l* = *s* depending on whether the incidence is from the fluid (*f*) side or the substrate (*s*) side, and *θ* denotes the incident angle with respect to the normal. Similarly, for the case of transmission through the grating,


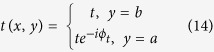


where


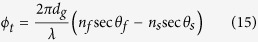


We approximate the incident beam from the laser by a normalized 2D Gaussian function 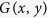. The diffraction grating object function *p*(*x,y*) is then,


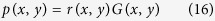


In our experimental setup, *b* (the diameter of the incident beam) = 1 mm; *λ* (wavelength) = 633 nm; and *D* (distance of the detection plane from the grating) = 1 m. Then, the Fresnel number *N*_F_ = 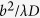
~0.4 which makes the Fraunhofer (far-field) approximation valid in our case^37^. The periodically modulated reflection and transmission coefficients will give rise to a diffraction pattern with diffracted efficiencies given by the standard diffraction integrals, which under the Fraunhofer approximation, amounts to taking the Fourier transform of the function *p*(*x, y*)^37^. For a normalized Gaussian function (unit input power), the diffraction efficiencies in R-mode for the zeroth and first orders, 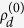 and 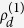 respectively, are given by,


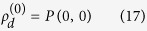



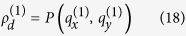


Here *P*(*q*_*x*_, *q*_*y*_) is the 2D Fourier transform of *p*(*x*,*y*) defined in Eq. (16) and 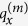 and 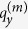 are coordinates in the conjugate space corresponding to the m^th^ diffracted order, i.e.


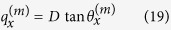



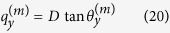


where,


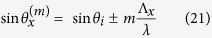



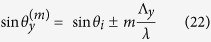


Here, *θ*_i_ is the incident angle on the grating (Fig. 4(b)) and Λ_*x*_ and Λ_*y*_ are the periodicities of the grating in the *x* and *y* directions, respectively. In our case, Λ_x_ = Λ_y_ = 10 μm. Similar expressions for the diffraction efficiencies of the zeroth (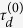) and first order (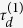) can be obtained by using *t*(x, y) in Eq. (16) instead of *r*(x, y). We use a multi-beam interference (MBI) model to derive relevant expressions for the DiOpter. We only consider the effect of a single reflection event, as the orders arising out of subsequent reflections are weaker. Also, as the optical path length of the phase grating is smaller than the illuminating wavelength (50 nm vs 633 nm), this grating can be considered as a “thin” grating wherein the first-order diffraction efficiency is larger than those corresponding to higher orders^38^.

The field associated with the detected first order signal in the R-mode (Fig. 4(b)) can be written as the superposition of three field components as,


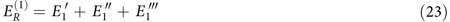


Eq. (23) can be written in terms of the diffraction efficiencies and Fresnel reflection and transmission coefficients, defined in the “Mathematical Modeling and Discussion” section, as


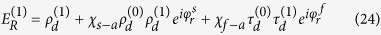


Similarly, in the T-mode


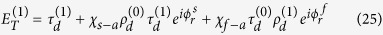


The detected first order intensities are the squared moduli of the field expressions. These were numerically evaluated using a MATLAB program which calculated the diffraction efficiencies described by Eqs. (17) and (18).

## Polyelectrolyte layer assembly and kinetic analysis of adsorption

**Figure 3:**
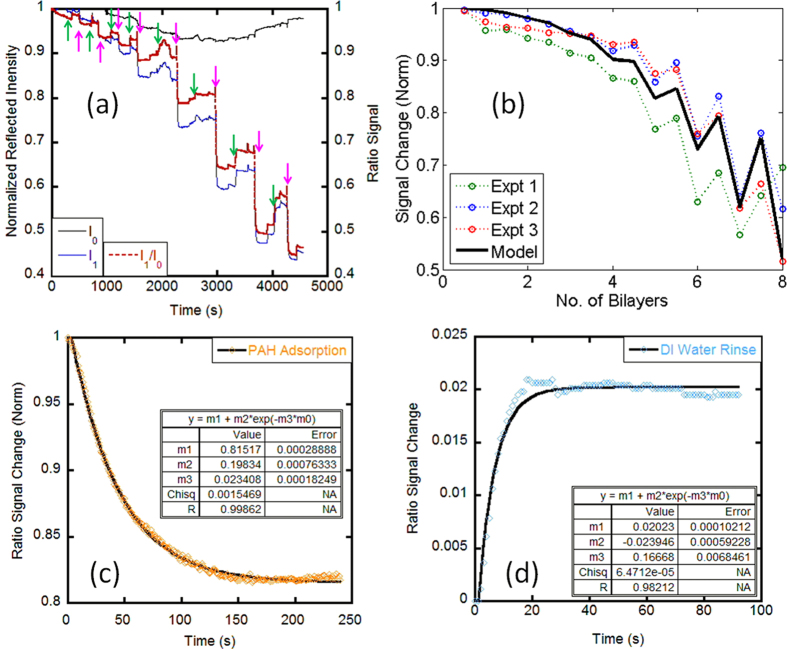
(**a**) Measurement of layer-by-layer growth of polyelectrolyte films comprising of PAH and PAA. The arrows indicate the addition of the polymer solution to the sensor alternating between PAH (green arrows) and PAA (pink arrows) to get a total of 8 bi-layers (**b**) Shows the repeatability of surface adsorption measurements. The MBI model closely matches the experimental results. (**c,d**) Shows the ability to obtain time-resolved adsorption data which is useful for estimating the kinetic parameters of surface reactions.

**Figure 4:**
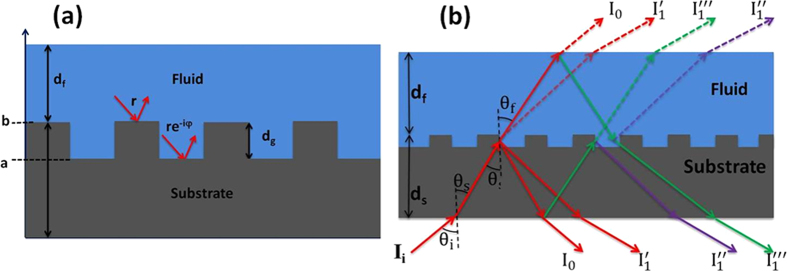
Ray diagram of multiple reflections in the DiOpter sensor illustrating the interfering field components I1′, I1″ and I1″′ generating the detected first order signal I1.

**Figure 5:**
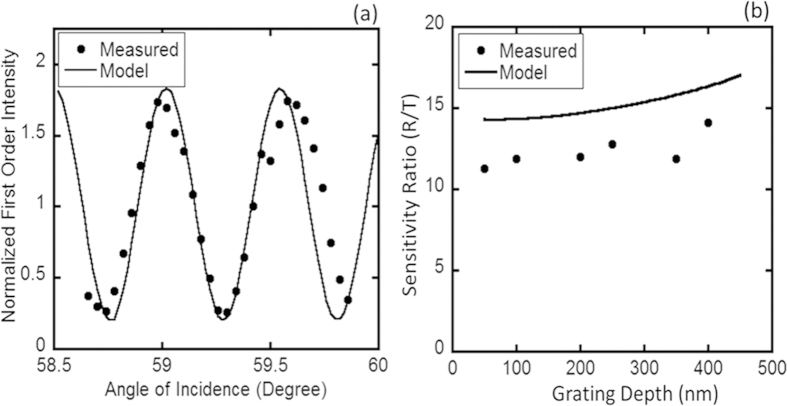
Validation of the DiOpter multi-beam interference model. (**a**) Shows the the modeled as well as the measured variation of the detected first order signal (I1) as a function of the incident angle. (**b**) Shows the ratio of R- to T- mode sensitivities estimated by the MBI model and measured with our setup over a wide range of grating depths.

We used cationic polyelectrolyte poly(allylamine hydrochloride) (PAH, Mw ~15,000 Da, Sigma Aldrich) and anionic polyelectrolyte poly(acrylic acid) (PAA, Mw ~2,00,000 Da, Sigma Aldrich). Materials were used as received without further purification. The two polyelectrolytes were alternately introduced into the flow cell using the sample injection system described previously. A DI water rinse step was introduced in between polyelectrolyte deposition steps to remove loosely bound molecules. A total of 8 bilayers (16 layers) were deposited. The observed zeroth and first order intensities were recorded during the entire deposition period leading to the data shown in Fig. 3(a) and 3(b). For the kinetic analysis, we extracted the data corresponding to a single adsorption or desorption step and normalized the recorded data with respect to the initial signal (t = 0). An exponential function *y* = *A* + *Be*^−*kt*^ was fitted to the normalized data to estimate the rate constant *k*. The characteristic time scale of adsorption (desorption) is *1/k*.

## Additional Information

**How to cite this article**: Kumawat, N. *et al.* Diffractive Optical Analysis for Refractive Index Sensing using Transparent Phase Gratings. *Sci. Rep.* **5**, 16687; doi: 10.1038/srep16687 (2015).

## Supplementary Material

Supplementary Information
