# Supplementary material for: Diffractive Optical Analysis for Refractive Index Sensing using Transparent Phase Gratings
Source: Sci Rep. 2015 Nov 18;5:16687. doi: 10.1038/srep16687 (PMC4649337; doi:10.1038/srep16687)
Supplement: Supplementary Information [file srep16687-s1.pdf]

Diffractive Optical Analysis for Refractive Index Sensing using Transparent Phase Gratings

Supplementary Information

Nityanand Kumawat, Parama Pal, Manoj Varma

Numerical calculation of terms in Eq. (2) and (3) of the main manuscript

Here we provide the numerical orders of the terms in the multi-beam interference (MBI) model discussed in the main text leading to the simplification of Eqs. (2) and (3) to Eqs. (5) and (6) respectively. The experimental parameters used in the calculations were: incidence angle θ (in air) = 60°, TM polarization, grating depth d_g = 250 nm, fluid layer depth d_f = 50 microns, substrate refractive index n_s = 1.5, fluid/sample refractive index n_f = 1.333. These parameters gave a good match to the experimentally obtained data as shown in Fig. 5 of the main manuscript. The Fresnel reflection coefficients χ_{s-a} and χ_{f-a} were calculated to be 0.031 and 0.0185 respectively. The Fresnel reflection coefficients are,

$$\chi_{s-a} = \frac{n_s \cos\theta_s - n_a \cos\theta_a}{n_s \cos\theta_s + n_a \cos\theta_a} \quad and \quad \chi_{f-a} = \frac{n_f \cos\theta_f - n_a \cos\theta_a}{n_f \cos\theta_f + n_a \cos\theta_a}$$

As shown in Fig. S1, the second term of Eq. (2) is 3 orders of magnitude smaller than the first and the third terms (which are comparable) enabling us to simplify Eq. (2) of the main text to Eq. (5) by neglecting the second term.

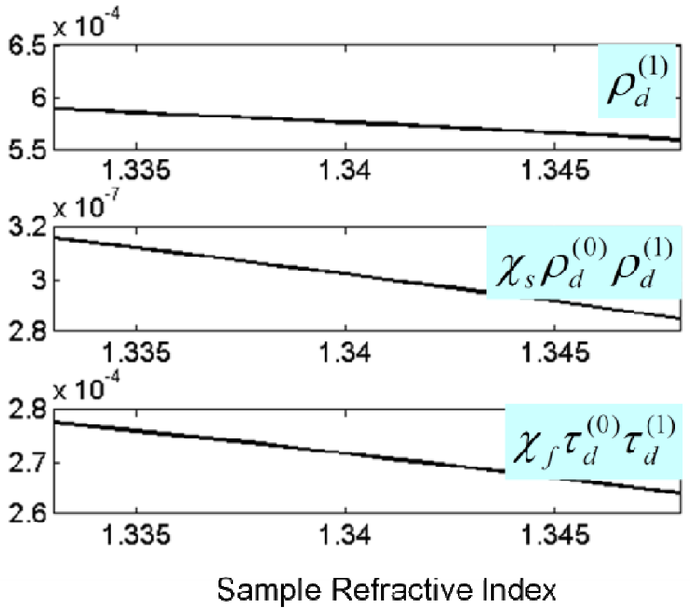

Fig. S1, Numerical values of the terms of Eq. (2) of the main text. Second term, i.e. the product of reflected diffracted efficiencies is negligible compared to the other two terms.

Similarly, Fig. S2 shows the numerical values of the terms in Eq. (3) of the main text. As we can see, the second and third terms are nearly four orders of magnitude smaller than the first term leading to the Eq. (6) in the main text.

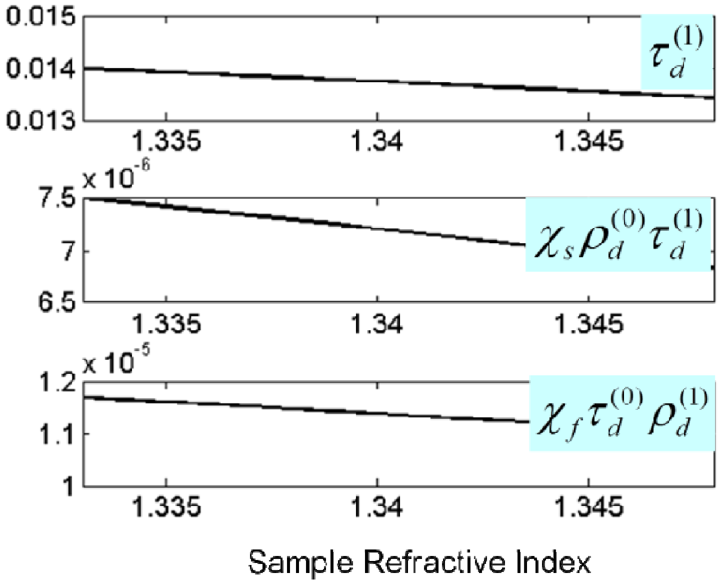

Fig. S2, Numerical values of the terms of Eq. (3) of the main text. Only the first term is significant and the other two terms can be neglected.

Numerical calculation of the amplification factor

Using the parameters listed in the previous section, the numerical values of each of the terms in Eq. (9) of the main text were calculated. These are shown in Fig. S3 from which we see that the term involving the factor A defined in Eq. (10) of the main text is the dominant term determining the sensitivity. This term is nearly two orders of magnitude higher than the remaining terms (fig. S3). This means that the depth of the fluid layer, relative to the wavelength, acts as an amplification factor improving the sensitivity and consequently the refractive index detection limit for the DiOpter. However due to the rapid variation of the $\sin(A\, n_f cos\, \theta_f)$ term as a function of incident angle with increasing A, ensuring the optimal operating point of $\sin(A\, n_f cos\, \theta_f) = 1$ requires very precise alignment of the incident beam.

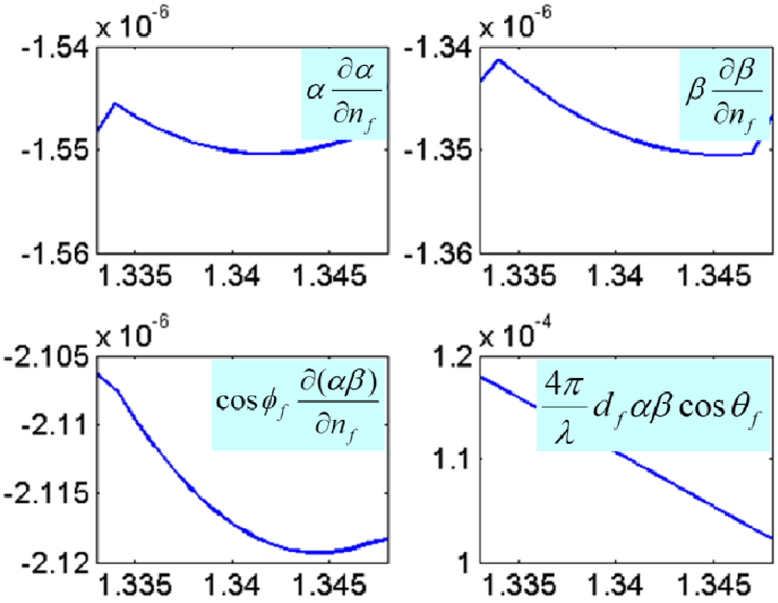

Fig. S3, Comparison of the numerical order of terms in Eq. (9). The x-axis in each figure is the sample refractive index

To determine the maximum sensitivity in R-mode, we set $\sin(An_f \cos\theta_f) = 1$ and compute $\eta_{R_{max}}^{(1)}$ by considering only the last term in Eq. (9). This yields a sensitivity of around 760/RIU while $\eta_T^{(1)}$ is only around 7.5/RIU (Fig. S4). The two order of magnitude difference between R-mode and T-mode sensitivity is precisely because of the two order of magnitude higher value of A compared to the other terms in Eq. (9).

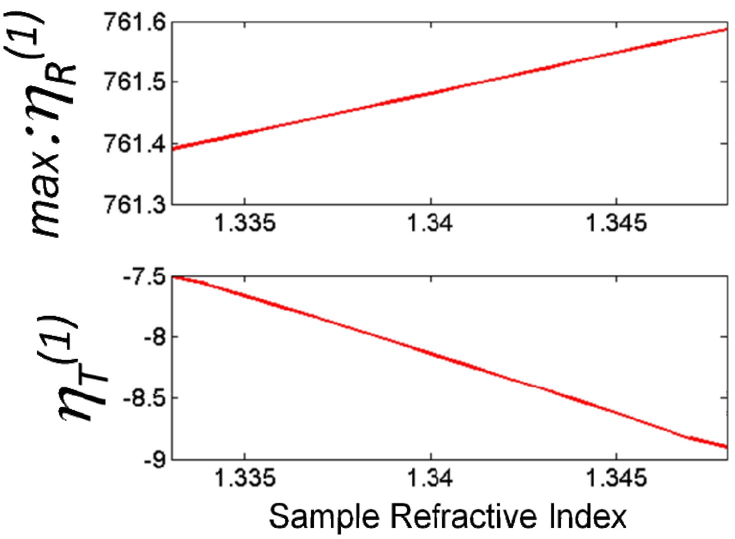

Fig. S4, Comparison of sensitivity of transmission mode measurement to the maximum sensitivity of the reflection mode measurement. Sensitivity of transmission mode is about 7.5/RIU (750%/RIU) while the maximum reflection mode sensitivity is around 750/RIU.
